# Predicting the functional consequences of non-synonymous single nucleotide polymorphisms in *IL8* gene

**DOI:** 10.1038/s41598-017-06575-4

**Published:** 2017-07-26

**Authors:** Tikam Chand Dakal, Deepak Kala, Gourav Dhiman, Vinod Yadav, Andrey Krokhotin, Nikolay V. Dokholyan

**Affiliations:** 1 Department of Biosciences, Manipal University Jaipur, Dehmi Kalan, Off Jaipur-Ajmer Expressway, Jaipur, 303007 Rajasthan India; 2 University Institute of Biopharma Sciences, Chandigarh University, Mohali, 140413 Punjab India; 3 Department of Microbiology, Central University of Haryana, Mahendergarh, 123029 Haryana India; 4 Department of Biochemistry and Biophysics, University of North Carolina, Chapel Hill, NC 27599 USA

## Abstract

Here we report an *in-silico* approach for identification, characterization and validation of deleterious non-synonymous SNPs (nsSNPs) in the interleukin-8 gene using three steps. In first step, sequence homology-based genetic analysis of a set of 50 coding SNPs associated with 41 rsIDs using SIFT (Sorting Intolerant from Tolerant) and PROVEAN (Protein Variation Effect Analyzer) identified 23 nsSNPs to be putatively damaging/deleterious in at least one of the two tools used. Subsequently, structure-homology based PolyPhen-2 (Polymorphism Phenotyping) analysis predicted 9 of 23 nsSNPs (K4T, E31A, E31K, S41Y, I55N, P59L, P59S, L70P and V88D) to be damaging. According to the conditional hypothesis for the study, only nsSNPs that score damaging/deleterious prediction in both sequence and structural homology-based approach will be considered as ‘high-confidence’ nsSNPs. In step 2, based on conservation of amino acid residues, stability analysis, structural superimposition, RSMD and docking analysis, the possible structural-functional relationship was ascertained for high-confidence nsSNPs. Finally, in a separate analysis (step 3), the IL-8 deregulation has also appeared to be an important prognostic marker for detection of patients with gastric and lung cancer. This study, for the first time, provided in-depth insights on the effects of amino acid substitutions on IL-8 protein structure, function and disease association.

## Introduction

Single nucleotide polymorphisms (SNPs) represent the most common type of genetic variation in humans^1^. Identification of single nucleotide polymorphisms having implications in inherited human diseases is among major challenges in human and medical genetics. Genetic variation caused by these SNPs, in particular non-synonymous SNPs (nsSNPs), occurring in protein coding regions alter the encoded amino acid at mutated site and may cause structural and functional changes in the mutated protein. However, not all such structural and functional changes due to nsSNPs are potentially damaging or deleterious. Some nsSNPs affect structural properties, while others show functional consequences. Additionally, some nsSNPs may be associated with a disease condition but others may not be related with any diseased phenotype, and are therefore, considered to be neutral. Functional consequences of any nsSNP, to a large extent, are based on attributes of the polymorphism^2^. Some attributes depend only on the sequence information, for example the types of residue found at the SNP location^2^. Therefore, it is very important to use an appropriate computational approaches and empirical rules based on probabilistic and machine learning to facilitate the discrimination of deleterious nsSNPs from neutral ones. In this work, we aimed to predict the structural and functional consequences of nsSNPs mapped in genetic variants of human interleukin-8 (*IL8*) gene.

The human interleukin-8 (IL-8 or CXCL8) is a pro-inflammatory chemokine that belongs to the supergene family of CXC chemokines and have associated role in inflammatory and infectious diseases^3^. There are two main families of chemokines; one that includes IL-8 has first two cysteine residues in the protein sequence separated by one amino acid (CXC chemokines), while in other family the first two cysteines in protein sequence are adjacent (CC chemokines)^4, 5^. In humans, IL-8 is expressed and secreted by various cells such as neutrophils, monocyte, endothelial and epithelial cells. These cells participate in inflammatory response after being challenged with a stimulus or signaling molecule, such as lipopolysaccharides (LPS)^6, 7^. The role of IL-8 is to activate inflammatory (immune) cells and amplify the inflammatory response. IL-8 accomplishes this by acting as a chemotactic factor (or chemoattractant) and call leucocytes, such as neutrophils, from the peripheral blood to the site of inflammation in tissues^8^. IL-8 binds to the G-protein coupled receptors, CXCR1 and CXCR2, present on the surface of neutrophils and triggers cell signaling leading to neutrophils activation. Activated neutrophils release their granular content containing lytic enzymes that help in neutralizing the pathogen. In this way, IL-8 plays a key role in neutrophil-mediated innate immune response. For instance, human cells such as epithelial cells and mononuclear cells over-express *IL8* gene in response to bacterial infection, Phorbol 12–myristate 13–acetate (PMA), and LPS treatment^9, 10^. The over-expression results in directed migration of neutrophils to the epithelial cells as a part of innate immune response so as to discourage the pathogen invasion^9, 10^. Besides this, the *IL8* gene has been implicated in a number of chronic inflammatory and genetic diseases, and therefore, the *IL8* gene is consistently being focused in the field of human medical genetics^11–15^.

The genetic diseases caused by polymorphic *IL8* genetic variants are basically an outcome of two situations. First being the altered expression of *IL8* gene, which results from polymorphisms in the *IL8* gene promoter region and results in turn altered binding of transcription factors onto their respective consensus sequence (transcription factor binding sites) in the *IL8* gene promoter region. The altered level of expression of *IL8* gene controls the level of pro-inflammation response and is linked with several disease phenotypes^11–15^. The second situation is structural changes in receptor binding sites in IL-8 protein that affect binding of IL-8 to its receptors (CXCR1 and CXCR2) present on the surface of inflammatory cells. This situation arises due to genetic polymorphisms in the *IL8* gene coding region that affects IL-8 protein structure, in particular, its receptor binding site. These structural changes in receptor binding sites also influence IL-8 binding to its receptor, and as a consequence, affect IL-8 mediated cell signaling and activation of inflammatory cells. In this work, we focus on mapping and identification of non-synonymous genetic polymorphisms in the *IL8* gene coding region that are expected to cause structural and functional alterations in IL-8 protein that may affect IL-8 binding to its cognate receptors present on surface of inflammatory cells.

The human *IL8* gene is located on chromosome 4q12-q13^5^. In human, the *IL8* gene encodes IL-8 protein as a 99 amino acid long precursor protein, which eventually undergoes processing to form several active isoforms of IL-8 protein^16, 17^. The expression of *IL8* gene is regulated mainly by transcription factor, namely nuclear factor-κB (NF-κB) through tumor necrosis factor (TNF) and TNF receptor associated factor 6 (TRAF6)^18, 19^; however, consensus sequences for some other factors have also been traced on *IL8* gene promoter^18^. The polymorphisms in the *IL8* promoter region influence binding of these transcription factors onto *IL8* gene promoter region and thus affect the transcriptional expression of *IL8* gene. To date most of the genetic studies focused mainly on polymorphisms identified in *IL8* gene promoter region. For instance, a single nucleotide polymorphism (SNP) of T/A at 251 nucleotides position upstream to start codon (−251 T/A; rs4073) in the *IL8* gene has been shown to modulate the level of IL-8 after treatment with LPS^7, 20^. This polymorphism in the *IL8* gene has been found associated with several inflammatory diseases such as chronic and aggressive periodontitis; macular degeneration and bronchiolitis; and several type of cancers such as hepatocellular carcinoma, lung cancer, breast cancer, and gastric cancer^20–26^. Regulation of IL-8 mediated inflammatory responses against bacterial infection is a well-known etiological factor for gastric cancer^27^. A meta-analysis study suggested that *IL-8*–251 allele A > T polymorphism might be a risk factor for gastric cancer^27, 28^. The −251A allele susceptibility in development of low penetrance cancer was also found in a meta-analysis of 42 case control studies^26^. In an another case control study it was found that inflammatory bowel disease and colorectal cancer risk might be associated with polymorphism in IL-8 −251 T/A^29^. A cohort study in north Indian population has found an association between IL-8 −251 T/A polymorphism and risk of bladder cancer^30^. In a literature it was reported that the IL-8 −251 “AA” genotype and “A” allele is susceptible with higher risk for glioma^31^. Association between IL8 −251 T/A polymorphism and acne vulgaris infection was also found in a study population^32^. A cohort study suggested that SNP in promoter region of *IL8* gene might be associated with an increased risk for recurrent *Clostridium difficile* infection. Linhartova *et al*. (2013) studied the relationship of four polymorphisms in *IL8* gene with chronic (CP) and aggressive (AgP) periodontitis, an inflammatory disease that cause loss of connective tissue and destruction of alveolar bone^10^. These polymorphisms were mapped at chromosome coordinates rs2227532 (−845 T/C), rs4073 (−251 T/A), rs2227307 (+396T/G), and rs2227306 (+781C/T). The allele present in the *IL8* polymorphic variants determines susceptibility to CP and AgP^33^. The −251TA heterozygote and +396TT homozygote genotype have been found associated with increase susceptibility to CP^34, 35^.

So far, most of the genetic analysis has been conducted on SNPs present in the *IL8* gene promoter region and no study has been conducted on genetic analysis of SNPs in the coding region. Taking into account this consideration and the fact that IL-8 plays a central role in many infectious and inflammatory diseases, in the present study we aimed to predict the functional consequences of 50 SNPs associated with 41 rsIDs in the *IL8* coding region as reported in dbSNP database. The study will provide in-depth understanding in relation to the role of nsSNP on IL-8 protein structure that may have potential role in IL-8 binding to its receptors on inflammatory cells and in disease susceptibility. In current study, we for the first time showed a strong correlation between IL-8 deregulation and the survival rate in cancer patients. Through our study, IL-8 deregulation has also appeared to be an important prognostic marker for the detection of patients with gastric and lung cancer but not for breast and ovarian cancer. This implicate that IL-8 deregulation does not impact the patient’s survival rate in case of sex or gender-specific cancer such as breast and ovarian cancer that are common among females.

## Results

**Figure 1:**
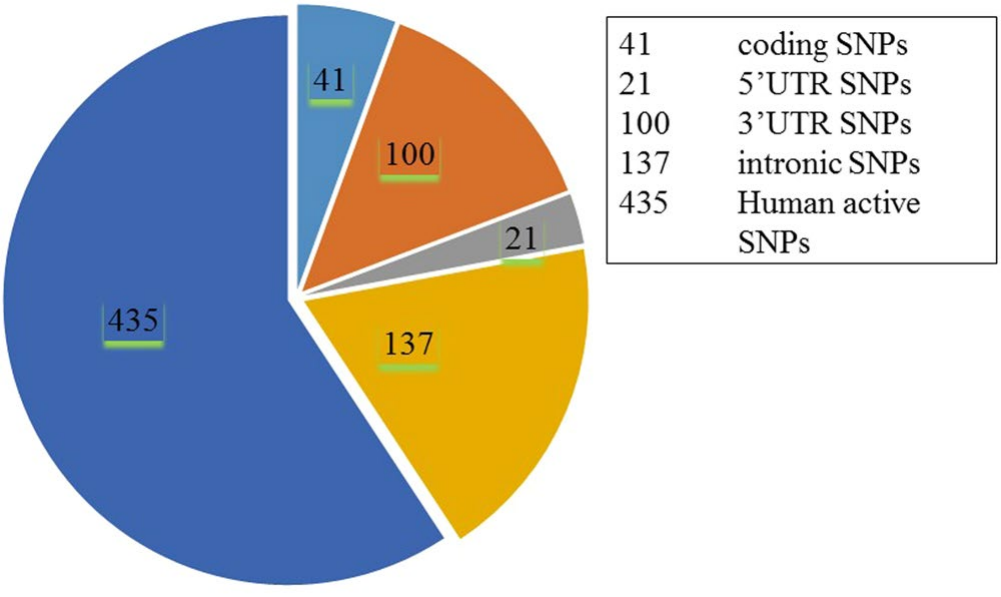
The pie-chart displays number of coding SNPs, 5′ UTR, 3′ UTR, intronic and other human active SNPs in human *IL8* gene (based on the dbSNP database).

All the reported SNPs of *IL8* gene were retrieved from NCBI dbSNP (http://www.ncbi.nlm.nih.gov/snp). A total of 734 SNPs were mapped in human *IL8* gene sequence. The numbers of SNPs in different functional class are reported in Fig. 1. In NCBI dbSNP database, 41 rsIDs were mapped that were associated with SNPs in the coding region. We also mapped 100 SNPs in the 3′ UTR, 21 in the 5′ UTR region, 137 in the intronic regions and the rest 435 SNPs were other human active SNPs but the current study focuses only on SNPs mapped in the coding region. Since, some rsIDs showed multiple SNPs at a single nucleotide position; we recorded 50 SNPs associated with 41 rsIDs in the *IL8* coding region, 3 synonymous and 47 non-synonymous (Table 1).

**Table 1:** A record of 50 SNPs, 3 synonymous and 47 non-synonymous, associated with 41 rsIDs in the *IL8* coding region.

rsID	Codons	SNP Type	Substitution
rs572157399	ATG-gTG	Nonsynonymous	*1 V
rs202071309	AAG-AcG	Nonsynonymous	K4T
rs563959935	CTG-CaG	Nonsynonymous	L5Q
rs200602609	GCC-tCC	Nonsynonymous	A6S
rs202202182	GCT-GaT	Nonsynonymous	A8D
rs564043731	CTC-tTC	Nonsynonymous	L9F
rs200254616	CTG-CaG	Nonsynonymous	L14Q
rs763622469	GAA-GAc	Nonsynonymous	E21D
rs751273843	GGT-aGT	Nonsynonymous	G22S
rs767339386	CCA-aCA	Nonsynonymous	P26T
rs199855020	AGT-cGT	Nonsynonymous	S28R
rs755727808	AAA-tAA	Nonsynonymous	K30*
rs138567132	GAA-GcA	Nonsynonymous	E31A
rs188378669	GAA-tAA	Nonsynonymous	E31*
rs188378669	GAA-aAA	Nonsynonymous	E31K
rs149273289	CAG-aAG	Nonsynonymous	Q35K
rs149273289	CAG-gAG	Nonsynonymous	Q35E
rs745916337	TAC-cAC	Nonsynonymous	Y40H
rs144469788	TCC-aCC	Nonsynonymous	S41T
rs749738011	TCC-TaC	Nonsynonymous	S41Y
rs200107073	CCT-CaT	Nonsynonymous	P43H
rs139503118	CAC-CgC	Nonsynonymous	H45R
rs774766411	AAA-gAA	Nonsynonymous	K50E
rs202114642	ATT-AaT	Nonsynonymous	I55N
rs202114642	ATT-AcT	Nonsynonymous	I55T
rs765951700	CCA-CtA	Nonsynonymous	P59L
rs373821605	CCA-tCA	Nonsynonymous	P59S
rs147544998	TGC-TGa	Nonsynonymous	C61*
rs147544998	TGC-TGt	Synonymous	C61C
rs140214046	GCC-aCC	Nonsynonymous	A62T
rs140214046	GCC-tCC	Nonsynonymous	A62S
rs758228010	ACA-cCA	Nonsynonymous	T64P
rs142957504	ACA-AgA	Nonsynonymous	T64R
rs759032011	AAG-AAa	Synonymous	K69K
rs759032011	AAG-AAc	Nonsynonymous	K69N
rs759032011	AAG-AAt	Nonsynonymous	K69N
rs762899923	CTT-CcT	Nonsynonymous	L70P
rs751369405	GAG-GAc	Nonsynonymous	E75D
rs536774132	TGT-TGa	Nonsynonymous	C77*
rs373408845	AAC-AAa	Nonsynonymous	N83K
rs780209935	TGG-TGa	Nonsynonymous	W84*
rs753921688	AGG-AaG	Nonsynonymous	R87K
rs756294837	AGG-AGa	Synonymous	R87R
rs756294837	AGG-AGc	Nonsynonymous	R87S
rs779068762	GTT-GcT	Nonsynonymous	V88A
rs779068762	GTT-GaT	Nonsynonymous	V88D
rs200662278	TTG-TaG	Nonsynonymous	L93*
rs185040023	AAG-AgG	Nonsynonymous	K94R
rs200005090	GCT-GtT	Nonsynonymous	A96V
rs201643630	TAA-TtA	Nonsynonymous	*100 L

**Figure 2:**
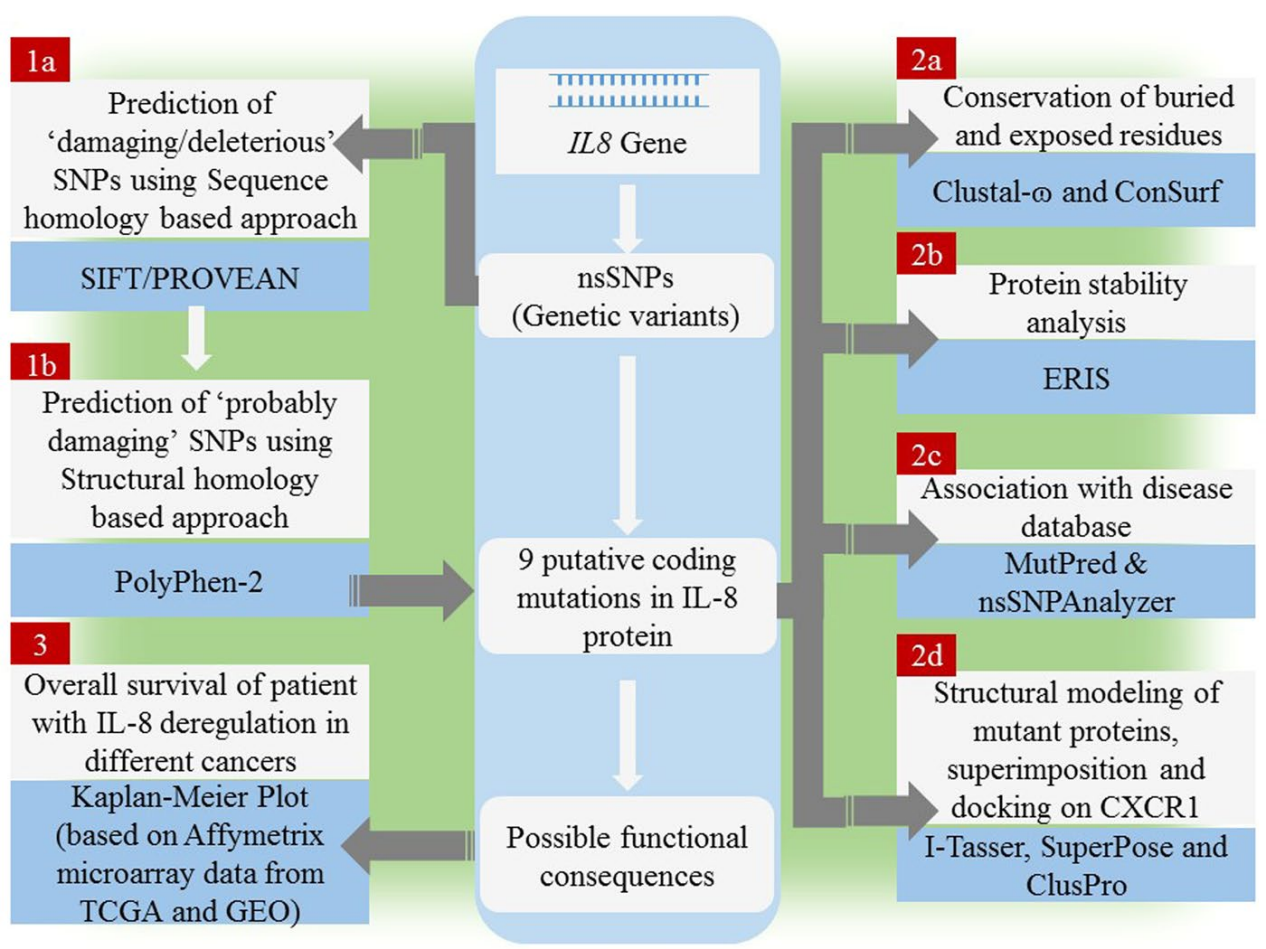
An overview of the experimental design for in-silico identification/characterization (step 1), validation (step 2) of nsSNPs in IL8 gene and associating deregulation of IL8 gene with survival of cancer patients (step 3).

For ascertaining the structural and functional consequences of 50 SNPs on IL-8 protein structure, we used a multi-tier approach comprising of three steps. The objective of the step 1 is to collect a set of high confidence SNPs in *IL8* gene. The step 1 has been performed in two sub-steps where the 50 SNPs mapped in *IL8* gene are first subjected to sequence homology based SNP prediction using SIFT (Sorting Intolerant from Tolerant) and PROVEAN (Protein Variation Effect Analyzer) (step 1a); and then structural homology based SNP prediction using PolyPhen-2 (step 1b). The objective of the step 2 is to validate high confidence SNPs (identified in step 1) using several different *in-silico* approaches such as stability analysis of mutant proteins using ERIS (step 2a), identification of disease associated SNPs using MutPred and nsSNPAnalyzer (step 2b), association of SNPs with highly conserved buried (structural) and exposed (functional) amino acid residues in IL-8 protein using Clustal Omega and ConSurf (step 2c), and comparison of tertiary structure of the modeled mutant proteins with the wild-type so as to infer possible structural-functional consequences of nsSNPs in IL-8 protein (step 2d). Finally, in step 3 we attempt to see if there exist any correlation between IL-8 deregulation and the survival rate of patients with different cancer types (breast, gastric, lungs and ovarian cancer) using the gene expression data obtained from clinical databases such as the cancer genome atlas (TCGA) and gene expression omnibus (GEO). For this, we used Kaplan-Meier Plot analysis. A flow chart explaining different steps and approaches used in current study is depicted in Fig. 2.

### Prediction and collection of a set of damaging nsSNPs using sequence and structure homology based SNP prediction

The step 1 aimed to predict and collect a set of damaging nsSNPs using sequence and structure homology based prediction algorithms. The step 1 is indeed a complex step where we performed: 1) sequence homology-based damaging SNP prediction using SIFT and PROVEAN (step 1a); and 2) structural homology-based probably damaging SNP prediction using PolyPhen-2 (step 1b). Getting a set of damaging SNPs predicted using only a single approach may not always be sufficient and useful, therefore, we used two tools (SIFT and PROVEAN) in step 1a so as to collect as much as possible SNPs for subsequent analysis in step 1b. The rationale behind using two tools is that since each tool has its own threshold cut-off value for SNP classification (as damaging and non-damaging) and this may sometimes results in false prediction for a SNP that score prediction value close to the threshold cut-off value. The use of two tools (instead of one), to some extent, resolve such biases in SNP classification and prediction. Furthermore, the sequence homology based approach and the structural homology based approach of SNP prediction are used in a complimentary way, wherein sequence homology based approach predicts SNPs using genetic point of view; whereas, the structural homology based approach predicts SNPs using functional point of view. The same combination has already been used in same manner previously by many other studies conducted by other research groups^36–38^. In fact, clinical laboratories also employ *in-silico* prediction tools, either alone or in combination, to predict missense single nucleotide variants of uncertain pathogenicity^39, 40^. Using SIFT/PROVEAN (step 1a) and PolyPhen-2 (step 1b) in a complementary way is expected to provide a set of ‘high-confidence’ damaging SNPs that are common in both approaches (means ‘damaging/deleterious’ prediction in at least one of the two webservers (SIFT or PROVEAN) used in step 1a as well as ‘probably damaging’ prediction (PolyPhen-2) in step1b.

### Prediction of damaging nsSNPs using SIFT and PROVEAN- sequence based approaches

Computational *in-silico* analysis using SIFT can predict 90% of damaging SNPs. Prediction of damaging effect of 50 SNPs mapped in genetic variants of *IL8* gene was done using SIFT (http://sift.jcvi.org/). SIFT algorithm predicts damaging and tolerated (non-damaging) substitutions based on a sequence homology and physical properties of sequence submitted. The functional consequences of amino acid substitutions caused due to nsSNPs were predicted and ascertained using the respective SIFT score. In accordance with Ng and Henikoff (2003) criteria, amino acid substitutions at a given position with normalized probabilities of ≤0.05 in a tolerance index are predicted to be damaging; whereas those with normalized probabilities ≥0.05 are predicted to be tolerated^41^. The lower the tolerance index, the higher functional impact a particular SNP is likely to have on encoded amino acid residue, and *vice versa*.

Three synonymous SNPs (sSNPs) associated with rsIDs, namely rs147544998 (TGC-TGt, C61C), rs759032011 (AAG-AAa, K69K), and rs756294837 (AGG-AGa, R87R), were not included in further *in-silico* analysis since these do not entail any change in amino acid in protein sequence. These 3 synonymous SNPs were scored as “tolerated” in our SIFT analysis. Out of 47 nsSNPs, 8 were nonsense nsSNPs and have functional consequence either start lost or stop gain or stop lost and were also not included in further analysis. The SIFT doesn’t score tolerated or damaging status for 8 nonsense SNPs that represented either start lost (*1 V), stop gain (K^30^*, E^31^*, C^61^*, C^77^*, W^84^*, L^93^*) or stop lost (*100 L) nsSNPs. Remaining 39 nsSNPs were missense nsSNPs and were either scored as “damaging” or “tolerated”. Among the 39 missense (non-synonymous) SNPs analyzed, 20 were scored as damaging while 19 were scored as tolerated in SIFT analysis. The outcome of the SIFT server has been shown in Table 2.

**Table 2:** Sequence homology-based prediction of damaging coding nsSNPs in *IL8* gene using SIFT and PROVEAN.

SNP rsID	Codons	Substitution	SNP Type	SIFT prediction		PROVEAN prediction	
				Prediction	Score	Prediction	Score
rs202071309	AAG-AcG	K4T	Nonsynonymous	Damaging	0	Neutral	−2.4
rs563959935	CTG-CaG	L5Q	Nonsynonymous	Damaging	0.01	Deleterious	−3.14
rs202202182	GCT-GaT	A8D	Nonsynonymous	Damaging	0	Deleterious	−3.66
rs200254616	CTG-CaG	L14Q	Nonsynonymous	Damaging	0	Deleterious	−4.63
rs763622469	GAA-GAc	E21D	Nonsynonymous	Damaging	0	Neutral	−1.47
rs199855020	AGT-cGT	S28R	Nonsynonymous	Damaging	0.04	Neutral	−1.13
rs138567132	GAA-GcA	E31A	Nonsynonymous	Tolerated	0.07	Deleterious	−4.96
rs188378669	GAA-aAA	E31K	Nonsynonymous	Damaging	0.01	Deleterious	−3.52
rs149273289	CAG-gAG	Q35E	Nonsynonymous	Damaging	0.04	Neutral	−2.17
rs749738011	TCC-TaC	S41Y	Nonsynonymous	Damaging	0	Deleterious	−4.27
rs139503118	CAC-CgC	H45R	Nonsynonymous	Tolerated	0.12	Deleterious	−5.83
rs202114642	ATT-AaT	I55N	Nonsynonymous	Damaging	0.02	Deleterious	−4.15
rs765951700	CCA-CtA	P59L	Nonsynonymous	Damaging	0	Deleterious	−9.35
rs373821605	CCA-tCA	P59S	Nonsynonymous	Tolerated	0.3	Deleterious	−7.21
rs758228010	ACA-cCA	T64P	Nonsynonymous	Damaging	0.01	Deleterious	−3.28
rs142957504	ACA-AgA	T64R	Nonsynonymous	Damaging	0.01	Deleterious	−3.53
rs759032011	AAG-AAc	K69N	Nonsynonymous	Damaging	0.01	Neutral	0.1
rs759032011	AAG-AAt	K69N	Nonsynonymous	Damaging	0.01	Neutral	0.1
rs762899923	CTT-CcT	L70P	Nonsynonymous	Damaging	0	Deleterious	−6.4
rs751369405	GAG-GAc	E75D	Nonsynonymous	Damaging	0.05	Neutral	−2.02
rs756294837	AGG-AGc	R87S	Nonsynonymous	Damaging	0.02	Neutral	−2.21
rs779068762	GTT-GcT	V88A	Nonsynonymous	Damaging	0	Deleterious	−3.4
rs779068762	GTT-GaT	V88D	Nonsynonymous	Damaging	0	Deleterious	−5.6

In order to increase the confidence level of prediction, the estimation of deleterious effect of 50 SNPs mapped in genetic variants of *IL8* gene was carried out using an additional web server namely PROVEAN (Protein Variation Effect Analyzer) (http://provean.jcvi.org). PROVEAN also works on a sequence based prediction algorithm^42^. The 3 synonymous SNPs that were scored as “tolerated” in SIFT analysis were scored as “neutral” in PROVEAN analysis also. PROVEAN also didn’t not scored the prediction for the 8 nonsense nsSNPs that represented start lost, stop gain, and stop lost. Out of 39 missense nsSNPs analyzed, 15 were scored as “deleterious” while 24 were scored as “neutral” in PROVEAN analysis (Table 2).

### Rationale behind using two tools (SIFT and PROVEAN) in SNP prediction

The specific aim of using two web-servers for prediction of “damaging” or “deleterious” nsSNPs was to increase the confidence level of prediction analysis and to obtain a subset of nsSNPs that were predicted to be “damaging” or “deleterious” in both SIFT and PROVEAN analysis. Getting a set of damaging SNPs predicted using only a single approach may not always be sufficient and useful because some SNPs that score close to threshold cut-off value are prone to false prediction. The use of two tools, to some extent, resolve such biases in SNP classification and prediction. All such nsSNPs that are predicted to be damaging/deleterious by at least one of the two tools used were subjected to further analysis by structure homology based approach, PolyPhen-2. In this way, we increased the number of candidate nsSNPs that represent the causative mutations within the protein coding region and may have possible implication in disease phenotype. We found 12 missense nsSNPs that were scored as either “damaging” or “deleterious” in both analyses. The corresponding amino acid substitution for these missense nsSNPs in IL-8 protein sequence were L5Q, A8D, L14Q, E31K, S41Y, I55N, P59L, T64P, T64R, L70P, V88D, and V88A. However, some nsSNPs were predicted to be damaging in SIFT analysis; however, found neutral in PROVEAN and *vice-versa* (Supplemental Table 1). There were 11 such nsSNPs that entail amino acid substitutions such as K4T, E21D, S28R, E31A, Q35E, H45R, P59S, K69N (both AAG-AAc and AAG-AAt), E75D, and R87S. The subset of nsSNPs comprising 12 “damaging” or “deleterious” nsSNPs common to both SIFT and PROVEAN analysis and 11 nsSNPs that resulted in damaging/deleterious prediction in only one of the two analyses (SIFT and PROVEAN) were chosen for further *in-silico* analysis using PolyPhen-2.

Both servers (SIFT and PROVEAN) are species independent (can be used for humans as well as for other mammals such as cow, buffalo, and pig) and also gene-independent (can be used for any gene encoding a protein (UGT1A1), enzymes (cytochrome P450), transcription factors, cytokines TGF-β etc.)^15, 38, 43^. Similarly, other servers used by us also species and gene-independent. Therefore, using these two servers for prediction of functional consequences of non-synonymous mutation in *IL8* gene seems to be scientifically logical.

### Prediction of damaging missense nsSNPs using PolyPhen-2- sequence based approaches

The subset of 23 missense nsSNPs that were found “damaging” or “deleterious” and incongruent in SIFT and PROVEAN analysis were subjected to functional analysis using PolyPhen-2. The subset of 23 missense nsSNPs was subjected to PolyPhen-2 analysis to predict the damaging nsSNPs in three different categories: probably damaging, possibly damaging, and benign (means tolerant). Among the amino acid substitutions correspond to 23 missense nsSNPs analyzed, 9 amino acid substitutions (K4T, E31A, E31K, S41Y, I55N, P59L, P59S, L70P and V88D) were scored as probably damaging (score > 0.96), 10 amino acid substitutions (A8D, L14Q, E21D, S28R, H45R, T64P, T64R, K69N (both AAG-AAc and AAG-AAt), K69A and V88A) were scored as possibly damaging (score > 0.2 and <0.96), and 4 amino acid substitutions (L5Q, Q35K, E75D and R87S) was scored as benign (score < 0.2). The outcome of the PolyPhen-2 server has been shown in Table 3. The K4T substitution lies at the N-terminal region of the IL-8 protein that is lost during protein maturation. Rest 8 amino acid substitutions that were predicted to be ‘probably damaging’ in PolyPhen-2 analysis were considered as high confidence nsSNPs that may have potential structural-functional impact on IL-8 protein. These 8 high confidence nsSNPs were subjected to further validation analysis in step 2.

**Table 3:** Structural homology-based prediction of damaging coding nsSNPs using PolyPhen-2.

Substitution	Effect	Score	Sensitivity	Specificity
K4T	Probably damaging	0.964	0.78	0.95
L5Q	Benign	0.164	0.92	0.87
A8D	Possibly damaging	0.906	0.82	0.94
L14Q	Possibly damaging	0.917	0.81	0.94
E21D	Possibly damaging	0.952	0.79	0.95
G22S	Possibly damaging	0.518	0.88	0.9
S28R	Possibly damaging	0.662	0.86	0.91
E31A	Probably damaging	1	0	1
E31K	Probably damaging	1	0	1
Q35K	Benign	0.012	0.96	0.78
S41Y	Probably damaging	0.999	0.14	0.99
H45R	Possibly damaging	0.549	0.88	0.91
I55N	Probably damaging	0.998	0.27	0.99
P59L	Probably damaging	1	0	1
P59S	Probably damaging	1	0	1
T64P	Possibly damaging	0.939	0.8	0.94
T64R	Possibly damaging	0.884	0.82	0.94
K69N	Possibly damaging	0.549	0.88	0.91
L70P	Probably damaging	0.997	0.41	0.98
E75D	Benign	0.012	0.96	0.78
R87S	Benign	0.072	0.94	0.84
V88A	Possibly damaging	0.856	0.83	0.93
V88D	Probably damaging	0.992	0.7	0.97

### Validation of damaging effect of 8 high confidence nsSNPs using multiple approaches

The objective of the step 2 is to validate high confidence SNPs (identified in step 1) at using different approaches one by one (step 1- step 2a; step 1- step 2b; step 1- step 2c; step 1- step 2d) as depicted in Fig. 2. In step 2 of our work, we subjected the high confidence SNPs (identified in step 1) to four different *in-silico* approaches such as stability analysis of mutant proteins using ERIS (step 2a), identification of disease associated SNPs using MutPred and nsSNPAnalyzer (step 2b), association of SNPs with highly conserved buried (structural) and exposed (functional) amino acid residues in IL-8 protein using Clustal Omega and ConSurf (step 2c), and comparison of tertiary structure of the modeled mutant proteins with the wild-type so as to infer possible structural-functional consequences of nsSNPs in IL-8 protein (step 2d).

### Effect of amino acid substitutions on mutant protein stability

It is widely known that the majority of disease associated nsSNPs affect the stability of the protein. We studied the effect of amino acid substitutions on the mutant protein stability using ERIS server. The server performs the structure based analysis of the mutant protein with substitution at a single amino acid residue and provides an estimation of free energy change in mutant protein with amino acid substitution at single site. The 8 amino acid substitutions, predicted to be ‘probably damaging’ in PolyPhen-2, were submitted to ERIS server to predict the ΔΔG stability (Table 4). We predicted ΔΔG with Eris using flexible backbone and with pre-relaxation setting because appropriate modeling of backbone of modeled proteins (as in flexible backbone setting) can significantly improve the free energy prediction by ERIS. We found that out of 8 mutants submitted to analysis, four mutant proteins (IL-8 E31A, IL-8 S41Y, IL-8 P59L, and IL-8 P59S) were predicted to have stabilizing effect while other four (IL-8 E31K, IL-8 I55N, IL-8 L70P, and IL-8 V88D) have destabilizing effect on the protein.

**Table 4:** Physical-principal based prediction of ∆∆G using Eris server (http://eris.dokhlab.org).

Models	Parameter used		Free energy (∆∆G) prediction	
	Backbone modeling	Pre-relaxation	Score	Mutation category
IL-8E31A	Flexible	Yes	−0.83	stabilizing
IL-8E31K	Flexible	Yes	0.97	destabilizing
IL-8S41Y	Flexible	Yes	−1.81	stabilizing
IL-8I55N	Flexible	Yes	3.64	destabilizing
IL-8P59L	Flexible	Yes	−3.07	stabilizing
IL-8P59S	Flexible	Yes	−1.67	stabilizing
IL-8L70P	Flexible	Yes	>10	destabilizing
IL-8V88D	Flexible	Yes	4.96	destabilizing

### Identification of disease phenotype associated with nsSNPs using MutPred and nsSNPAnalyzer

The same subset of 8 amino acid substitutions scored as ‘probably damaging’ was subjected to *in-silico* phenotypic analysis using nsSNPAnalyzer. The nsSNPAnalyzer is web-tool to predict whether a nsSNPs has a disease phenotype. The nsSNPAnalyzer also provides additional useful information about the SNP to facilitate the interpretation of results, for instance, structural environment, area buried, fraction polar, and secondary structure. The analysis using nsSNPAnalyzer requires information contained in the multiple sequence alignment and information contained in the input PDB 3D protein structure to make predictions. We used the crystal structure of IL-8 protein (PDB ID: 5d14) retrieved from the Protein Data Bank. The analysis using nsSNPAnalyzer predicted L70P amino acid substitution to be associated with disease phenotype. The amino acid substitution was found associated with rsIDs, rs762899923. The outcome of the nsSNPAnalyzer server has been shown in Table 5. We compared the results obtained from the nsSNPAnalyzer and that obtained from SIFT, PROVEAN and PolyPhen-2.

**Table 5:** Prediction of disease related amino acid substitution and phenotypes by nsSNPAnalyzer.

SNPs	Phenotype	Environment	AreaBuried	FracPolar	Secondstr
K4T	Unknown	—	—	—	—
E31A	Neutral	EC	0.094	0.896	C
E31K	Neutral	EC	0.094	0.896	C
S41Y	Neutral	EC	0.079	0.906	C
I55N	Neutral	B3S	0.509	0.719	S
P59L	Neutral	EC	0.016	0.854	C
P59S	Neutral	EC	0.016	0.854	C
L70P	Disease	B2S	0.61	0.323	S
V88D	Neutral	B1H	0.508	0.219	H

For the MutPred, the same subset of 8 amino acid substitutions scored as ‘probably damaging’ was subjected to *in-silico* phenotypic analysis was used. The MutPred is web-tool to predict the diseased phenotype and to identify the molecular mechanisms that result amino acid substitution caused by nsSNPs^44^. The MutPred also provides additional useful information such as gain or loss of solvent accessibility, molecular recognition features (MoRFs), stability, catalytic sites, and post-translation modification sites etc. that further aid in the interpretation of obtained results. Scores with g-value > 0.5 and p-value < 0.05 are referred to as actionable hypotheses, whereas the scores with g-value > 0.75 and p-value < 0.05 are referred to as confident hypotheses. The g-value and p-value scores for the 8 amino acid substitutions has been shown in Table 6. In MutPred prediction, the L70P and V88D substitution showed highest g-values and lower p-values. We found that the L70P amino acid substitution was also predicted as “damaging” in SIFT, PROVEAN and PolyPhen-2 analysis and “diseased” in nsSNPAnalyzer with high score (Tables 2, 3 and 5).

**Table 6:** Prediction of disease related amino acid substitution and phenotypes by MutPred.

SNPs	Actionable/Confident hypothesis	g-value	p-value
E31A	Loss of solvent accessibility	0.572	0.0404
E31K	Gain of MoRF binding	0.568	0.0031
S41Y	Gain of solvent accessibility	0.510	0.0739
I55N	Gain of disorder	0.710	0.033
P59L	Gain of catalytic residue at P59	0.609	0.051
	Loss of glycosylation at S57		0.0797
P59S	Loss of glycosylation at S57	0.521	0.0829
L70P	Loss of stability	0.771	0.0189
	Loss of catalytic residue at L70		0.0214
V88D	Gain of disorder	0.786	0.0306
	Loss of MoRF binding		0.0325
	Gain of ubiquitination at K91		0.0401

### Association of SNPs with highly conserved buried (structural) and exposed (functional) amino acid residues in IL-8 protein

From structural point of view, IL-8 expresses as a 99 amino acid long protein but its active and functional form contains only 72 residues that results from cleavage of first 27 amino acids at N-terminal. The monomeric unit in the three-dimensional structure reveals a highly flexible NH,-terminal region followed by three antiparallel β strands and a COOH-terminal α-helix.

**Figure 3:**
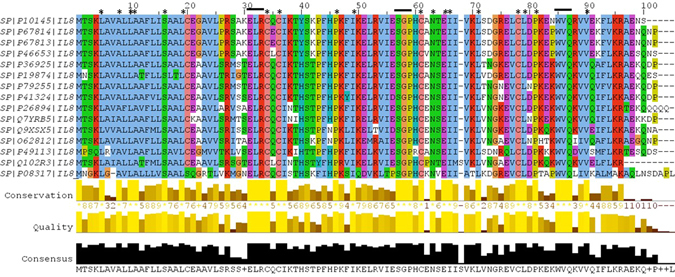
Amino acid alignment of human IL-8 protein (UniProt ID: P10145) along with its homologues in phylogenetically close species in mammals and fouls. Solid horizontal bars indicate conserved sequence motifs and residues with asterisk (*) mark indicate evolutionary conserved amino acids. The amino acid identities were colored according the Clustal color scheme, and the conservation index at each alignment position were provided by Jalview^61^.

**Figure 4:**
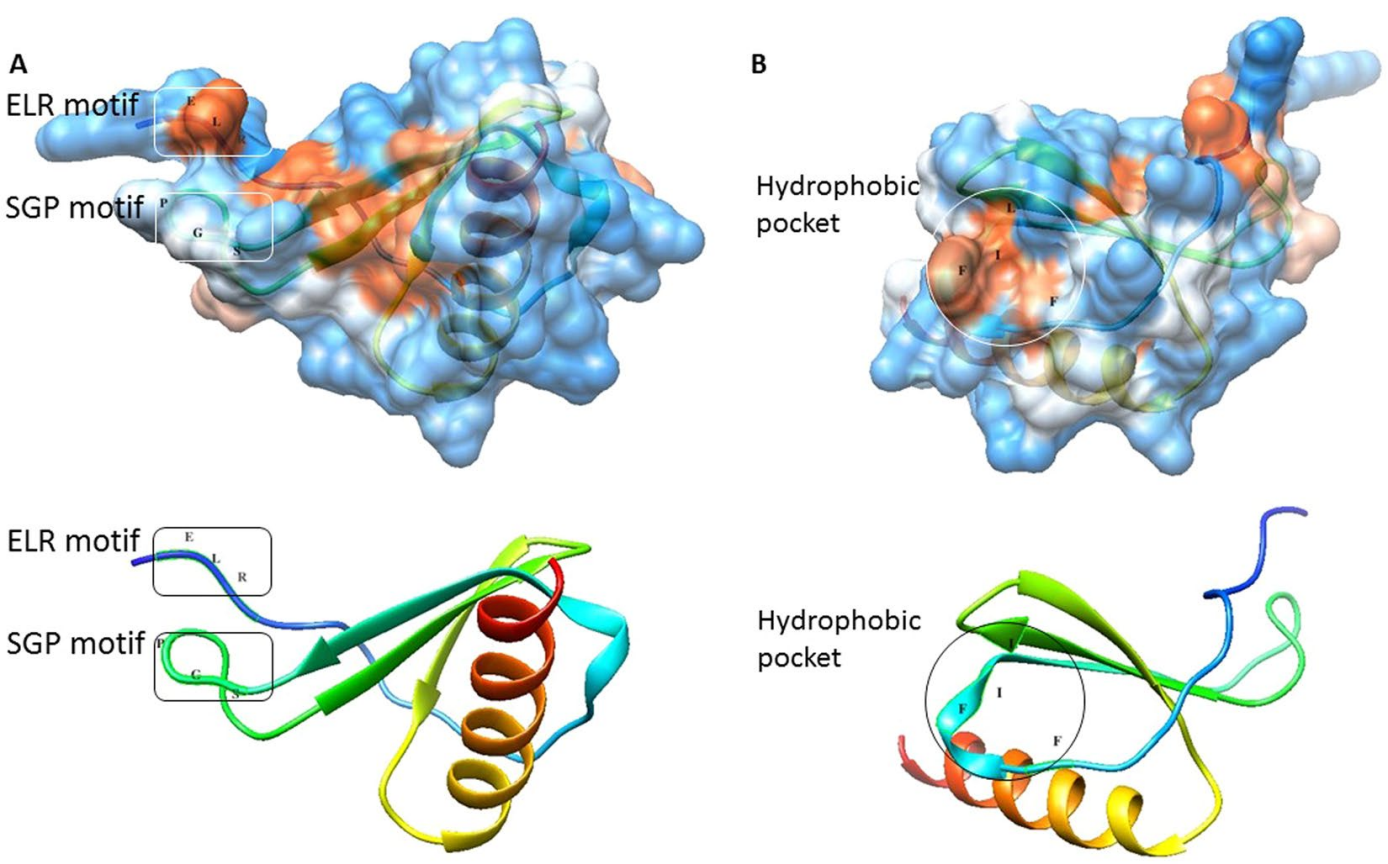
Structural model of modeled human IL-8, wherein panel A shows two conserved sequence motifs, ELR^31–33^ and SGP^57–59^, that face each other and form a structural scaffold putatively involved in IL-8 binding to its receptor; and panel B shows the hydrophobic pocket formed of F^44^, F^48^, I^49^ and L^70^ on the surface of IL-8 protein having role in receptor binding.

Sequence based structural-functional analysis of IL-8 was performed using Clustal Omega based multiple sequence alignment analysis. For this analysis, the IL-8 protein sequence (Uniprot ID: P10145) was retrieved from Uniprot Knowledgebase. The IL-8 protein sequence was BLASTed against the UniprotKB/SwissProt entries and aligned using Clustal Omega with default settings. The results generated by the Clustal Omega tool consist of IL-8 protein sequence aligned with other phylogenetically close sequences from other organisms (Fig. 3). The results contain a colorimetric conservation score in range of 1–10. Multiple sequence alignment using Clustal Omega revealed that human IL-8 protein sequence contains a number of conserved residues and motifs. The highly conserved amino acid residues in human IL-8 protein were L^5^, A^8^, L^10^, A^11^, S^16^, L^19^, I^37^, P^46^, I^49^, N^63^, E^65^, I^66^, L^70^, L^78^, P^80^, and V^89^. There are five conserved cysteine residues, four are involved in intramolecular disulfide bridge formation. The C^34^ and C^36^ are part of the C-X-C motif and respectively form C^61^ and C^77^ disulfide bridge^45^. Besides this, we mapped three small conserved motifs sequences, ELR^31–33^, SGP^57–59^, and WVQ^84–86^ (Figs 3 and 4 panel A). The N-terminal of IL-8 protein is involved in receptor binding and includes a receptor binding site, the ELR^31–33^ motif^45^.

**Figure 5:**
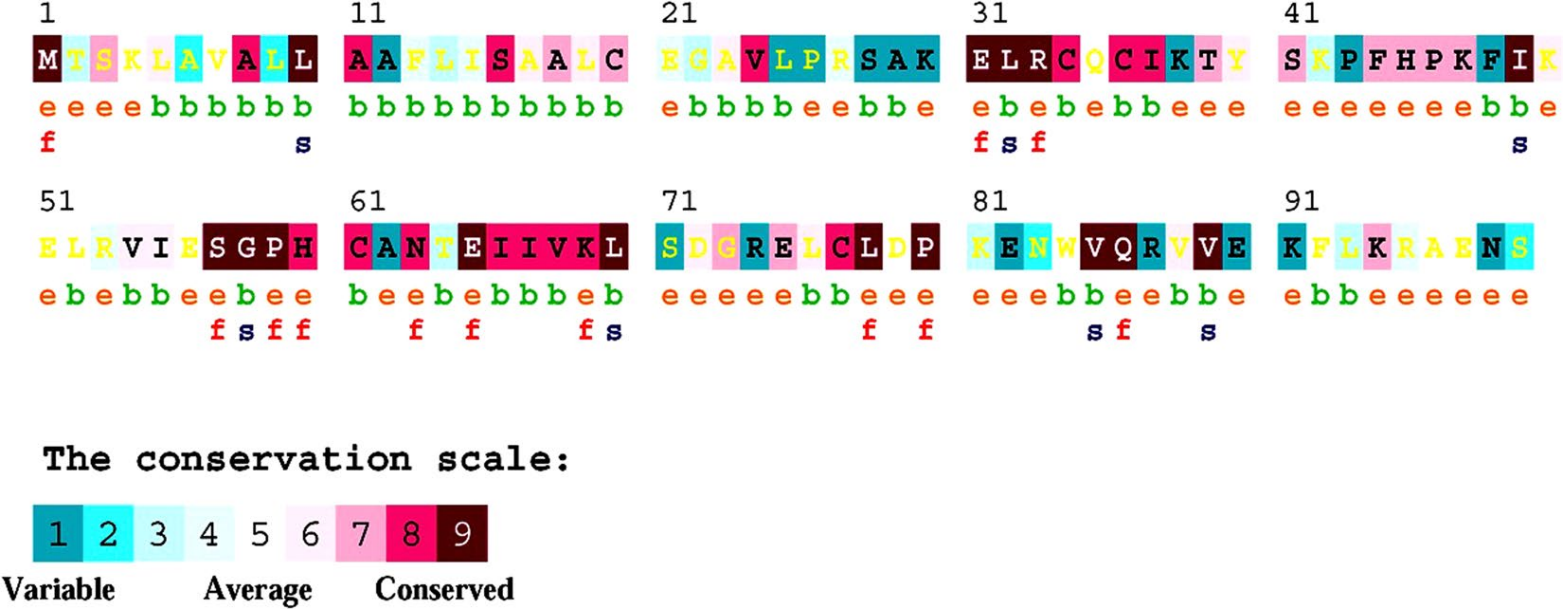
Consurf analysis of human interleukin-8 protein (Uniprot ID: P10145).

We predicted 9 amino acid substitutions in IL-8 protein to be ‘probably damaging’ based on PolyPhen-2 analysis. In our PolyPhen-2 analysis, K4T, E31A, E31K, S41Y, I55N, P59L, P59S, L70P and V88D were found to be ‘probably damaging’. The impact of K4T substitution on IL-8 structure and function has not been interpreted because this residue lies in the N-terminal of IL-8 that gets cleaved off during process of mature IL-8 formation. The E^31^ is an important residue that form the part of highly conserved ELR^31–33^ motif, which act as an epitope for receptor binding. ConSurf analysis identified conserved residues in IL-8 protein and predicted residues to be exposed or buried in the IL-8 protein structure (Fig. 5). ConSurf analysis predicted E^31^ to be exposed and conserved residue, i.e., a functional residue. ConSurf analysis also predicted R^33^ to be exposed and conserved residue, i.e., a functional residue. We assume that these charged (E^31^ and R^33^) residues facilitate electrostatic interaction between IL-8 and IL-8 receptors. If so, we suggest that E31A and E31K substitutions would also entail some structural change in the ELR^31–33^ motif that are expected to putatively affect IL-8 binding with its receptor, i.e., its function.

### Structure-based functional analysis

Next we compared the tertiary structure of the modeled mutant proteins with the wild-type so as to infer possible structural-functional consequences of nsSNPs in IL-8 protein (step 2d). We found that, the conserved ELR^31–33^ motif is immediately followed by conserved C-X-C motif and conserved I^37^ residue. These three structural features form a distinct region (a pocket) on the surface of IL-8 having role in receptor binding^46^. There is another distinct region, a hydrophobic pocket, in the IL-8 protein structure that comprises F^44^, F^48^, I^49^ and L^70^ and is also implicated in receptor binding (Fig. 4 panel B). Williams *et al*. (1996) through their mutagenesis study on IL-8 protein found that mutations in any residue belonging to these two regions (ELR^31–33^ epitope and hydrophobic pocket) on the surface cause no change in IL-8 protein structure but affects its receptor binding ability^46^. Williams *et al*. (1996) showed that Y^40^, F^48^, L^70^, S^71^ play important role in receptor binding^46^. Clark-Lewis *et al*. (1994) also demonstrated that G^58^ and P^59^ (residues belonging to SGP^57–59^ motif) are required for IL-8 activity in a functional study using a hybrid protein derived from IL-8 regions into IP10, a similar protein lacking IL-8 activity^45^. The C-terminal and the α-helical region spanning from N^83^ to E^97^ have no role in receptor binding^45, 47^.

**Figure 6:**
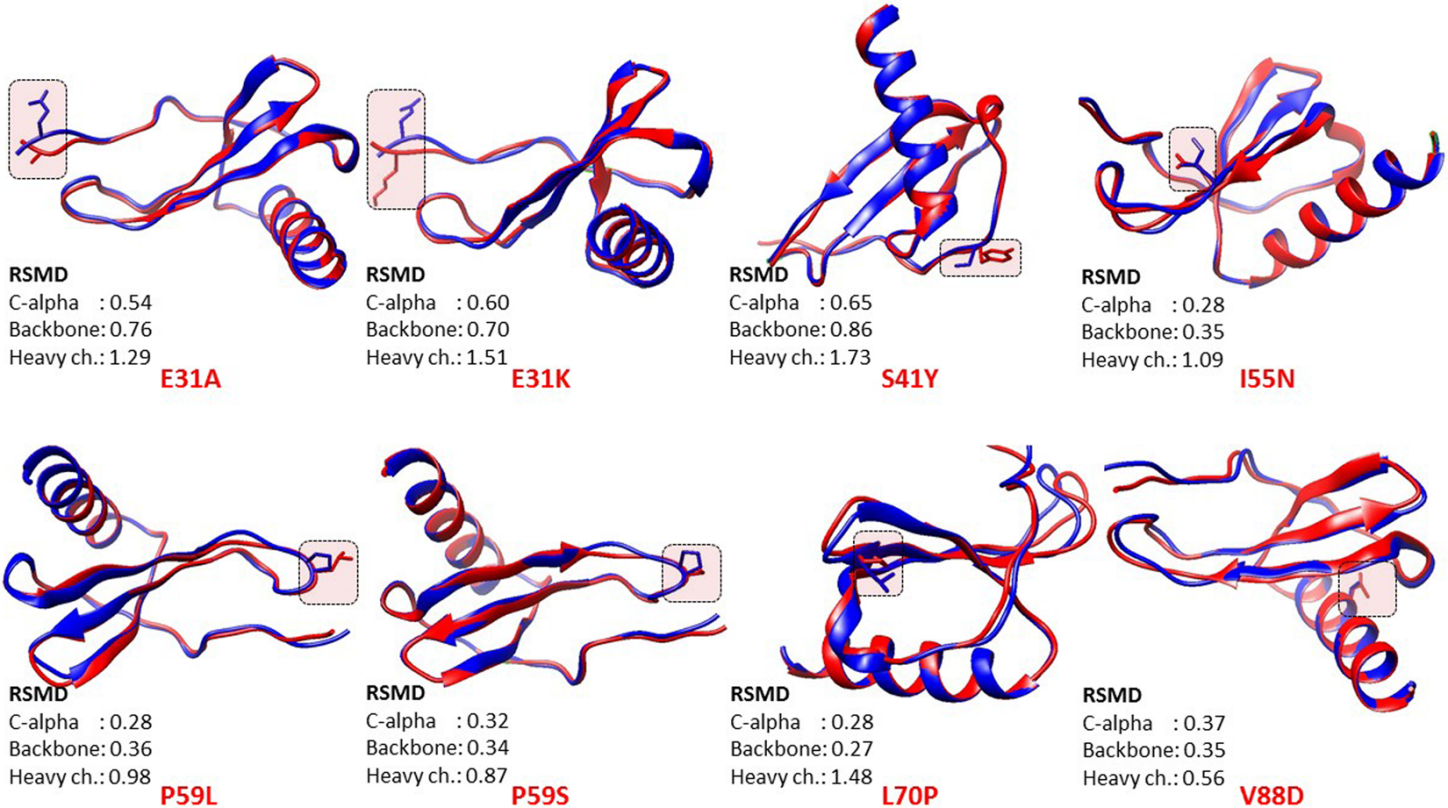
Structural superimposition of modelled mutant proteins (in pink) on the wild type IL-8 protein (in peacock blue) using PyMOL.

Our structure-based approach for studying effect of amino acid substitution in IL-8 protein showed that both E31A and E31K substitution resulted in only local changes that mainly changed the orientation of N-terminal end of the mature IL-8 protein, which is putatively involved in electrostatic interaction between IL-8 and its receptors (Fig. 6). In congruent with this mutation studies such as alanine substitution of ELR^31–33^ motif showed that the motif is indispensable for IL-8 activity^48^. Chemically synthesized analogs containing single residue replacement in the ELR^31–33^ motif were also found to be sensitive to modification^49^. ELR^31–33^ motif also plays an important role in cancer angiogenesis^50^. The P^59^ is also an important residue that forms the part of highly conserved SGP^57–59^ motif implicated in receptor binding by Clark-Lewis *et al*. (1994)^45^. ConSurf analysis predicted P^59^ to be exposed and conserved residue, i.e., a functional residue. Our structure based functional analysis revealed that conserved ELR^31–33^ motif and SGP^57–59^ motif face each and form a structural scaffold that is presumably involved in receptor binding. Besides this, the 3D structure of IL-8 has identified the SGP^57–59^ motif as an essential conformation referred to as SGP^57–59^ turn that brings C^34^ and C^61^ in close proximity and proper geometry to allow disulfide bridge formation^45^ (Fig. 4 panel A). The interactive hydrophobic surface view of IL-8 visualized in Chimera 1.10.1 (Fig. 4 panel A) showed that the SGP^57–59^ motif is relatively exposed in the IL-8 structure (wild type) suggesting its direct involvement in IL-8 receptor binding. We found that, both P59L and P59S, substitutions entail considerable structural change in the SGP^57–59^ motif locally as well as the region on both side of the SGP^57–59^ motif suggesting that these substitutions are expected to putatively affect IL-8 binding with its receptor. L^70^ is an important residue and is part of hydrophobic pocket (formed by F^44^, F^48^, I^49^ and L^70^) on the IL-8 surface. ConSurf analysis predicted L^70^ to be buried and conserved residue, i.e., a structurally important residue. Our structure based approach revealed that L70P substitution result in high root mean square deviation in mutant protein as compared to the wild type protein. We, therefore, suggest that L70P substitution putatively entail structural change in the hydrophobic pocket formed by F^44^, F^48^, I^49^ and L^70^ (Fig. 6). Using nsSNPAnalyzer, L70P substitution has been predicted to be associated with disease phenotype. In fact, majority of disease associated nsSNPs had been found to be located in surface pockets of protein structures^51^.

We superimposed the 8 mutant IL-8 proteins onto the PDB structure of the wild type protein using SuperPose ver 1.0 server (Fig. 6). We found that the substitutions (such as E31A and E31K) in the N-terminal receptor binding ELR^31–33^ motif of the IL-8 protein entail local conformation changes as well as changes in the turn that harbors SGP^57–59^ motif, which has also been implicated in IL-8 binding with its receptor CXCR1. The substitutions such as P59L and P59S involves the proline residue which is most commonly found in turn and helps in the formation of beta-turns in protein. Substitution of proline with other amino acids is thus expected to effect the conformation of the SGP^57–59^ turn in IL-8 protein. This is in congruent with the high heavy chain RSMD for IL-8 P59L and IL-8 P59S obtained in structural superimposition analysis (Fig. 6). For remaining four amino acid substitutions in IL-8 proteins, i.e., S41Y, I55N, L70P and V88D, our structural analysis showed that the former two amino acid substitutions entail considerable structural change in the local structure and the latter two amino acid substitutions led to structural change in IL-8 protein, both locally and throughout the protein structure.

### Molecular docking studies for studying the effect of E31A and E31K substitution on binding of IL8 to its receptor CXCR1

Based on our structure-based analysis, we hypothesized that E31A and E31K substitution may result in local changes that mainly changed the orientation of N-terminal end of the mature IL-8 protein, which is putatively involved in electrostatic interaction between IL-8 and its receptors. In order to support our hypothesis, we conducted docking studies on IL-8 E31A and IL-8 E31K mutant proteins with CXCR1 (PDB id: 2LNL). The docking was performed using ClusPro webserver using custom settings (https://cluspro.bu.edu/login.php)^75^. CXCR1 is a membrane receptor protein and the IL-8 is expected to bind it on the extracellular side. CXCR1 has seven transmembrane helices (TMHs); however, the transmembrane helices do not represent docking site for IL-8 protein, except the hydrophilic residues at the end of a TMH towards the extracellular side. On the contrary, the extracellular loops present between two adjacent TMHs could be the putative docking site for IL-8. Upon performing docking using default setting, we obtained mostly irrelevant docked poses in which the IL-8 wild type protein interacted with the transmembrane helices of the CXCR1. However, in a couple of poses, the C-terminal helix of IL-8 was found to interact with the extracellular side of the CXCR1. Previous studies showed that IL-8 protein contains the ELR^31–33^ and SGP^57–59^ motif that interact with CXCR1^45, 46^. Therefore, we used custom setting in the ClusPro and performed docking in which the attraction of the ELR^31–33^ and SGP^57–59^ residues of the IL-8 for the CXCR1 was specified. In order to specify attraction, we entered the residues corresponding to ELR^31–33^ and SGP^57–59^ for the ligand IL-8 in the entry field provided in the ClusPro for all docking experiment performed.

**Figure 7:**
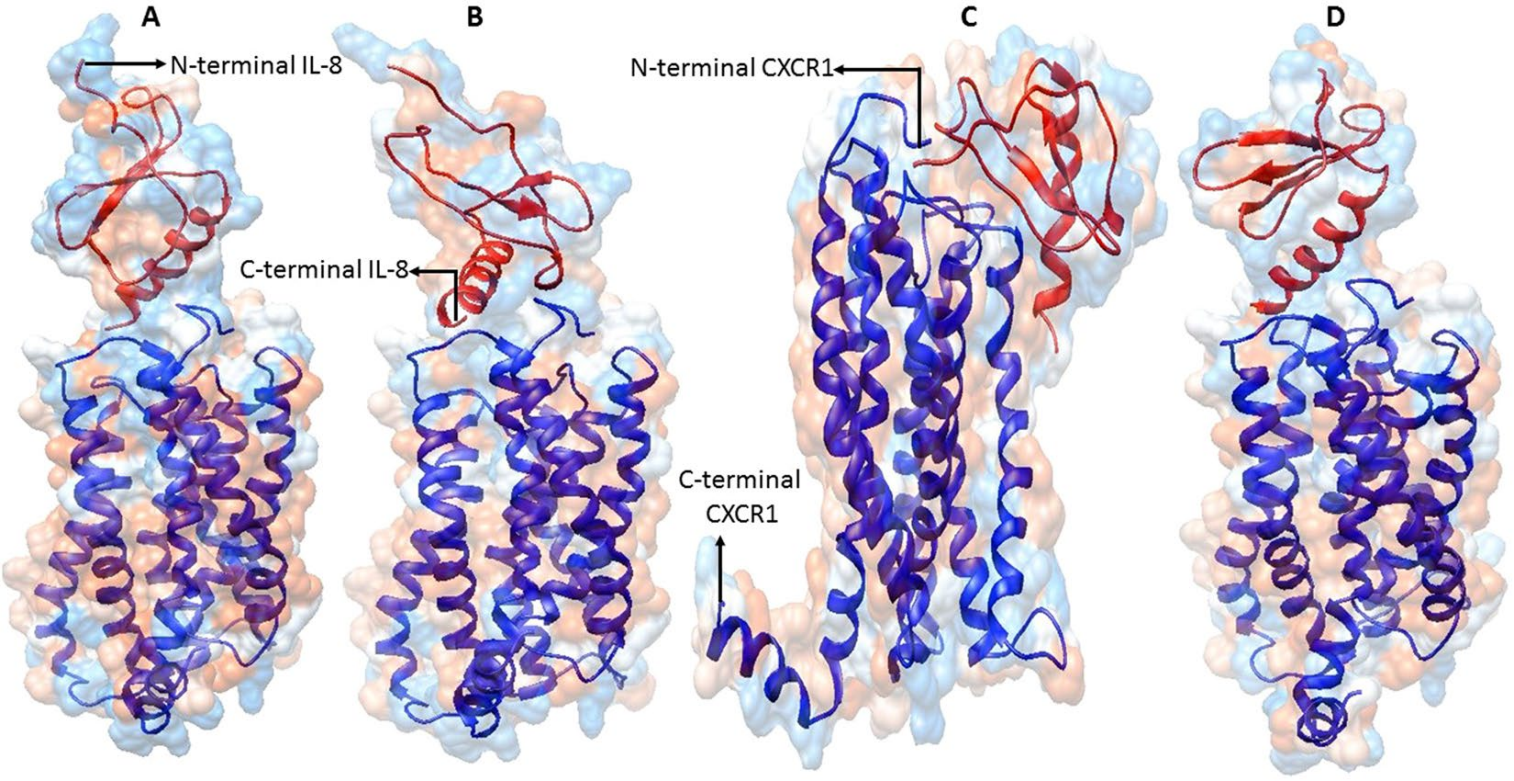
The representative docking poses of IL-8 wild type (IL-8 WT) (Panel A) and mutant proteins (IL-8 E31A & IL-8 E31K) (Panel B–D) onto its receptor IL-8R1 (PDB id: 2LNL). The IL-8 WT and mutants proteins are red colored and the receptor CXCR1 is blue colored. The N- and C-terminal ends of both IL-8 WT/mutants and CXCR1 are also marked.

For the IL-8 WT protein, entering the ELR^31–33^ and SGP^57–59^ residues in the attraction entry field resulted in ten most probable docked poses for each electrostatically-favored, hydrophobically-favored and Van der waals-electrostatically favored models. There was no single relevant docked pose in electrostatically favored and hydrophobically favored models suggesting that electrostatic and hydrophobic interaction alone are not sufficient for IL-8 interaction with its receptor CXCR1. However, in model that represented a combination of Van der waals- electrostatically favored models, there were six relevant poses that represented IL-8 docked onto CXCR1 on the extra-cellular side. All the six poses showed IL-8 interaction with its receptor CXCR1 through the C-terminal helix region (Fig. 7 panel A). For the IL-8 E31A mutant protein, entering the ELR^31–33^ and SGP^57–59^ residues in the attraction entry field resulted in nine out of ten docked poses to be relevant in Van der waals- electrostatically favored models (Fig. 7 panel B). This strongly suggests that E31A mutation in IL-8 has advantageous role in IL-8 interaction with its receptor CXCR1. This is in congruent with our ERIS based result wherein E31A mutation has been found to be stabilizing. On the contrary, one out of ten docked pose was also found to be relevant in electrostatically favored models (Fig. 7 panel C) and none found relevant in hydrophobically favored models. For the IL-8 E31K mutant protein, considering the ELR^31–33^ and SGP^57–59^ residues in the attraction field resulted in five out of ten docked poses to be relevant in Van der waals- electrostatically favored models (Fig. 7 panel D). This strongly suggests that E31K mutation in IL-8 does not have any beneficial role in IL-8 interaction with its receptor CXCR1. This is in congruent with our ERIS based result wherein E31K mutation has been found to be destabilizing. Additionally, there was not a single docked pose that represented relevant docking in both electrostatically favored and hydrophobically favored models. From our docking analysis it became imperative that besides electrostatic interaction, Van der waals forces may have important role in IL-8 binding with its receptor CXCR1.

### Clinical correlation between IL-8 deregulation and the survival rate of patients with different cancer types

**Figure 8:**
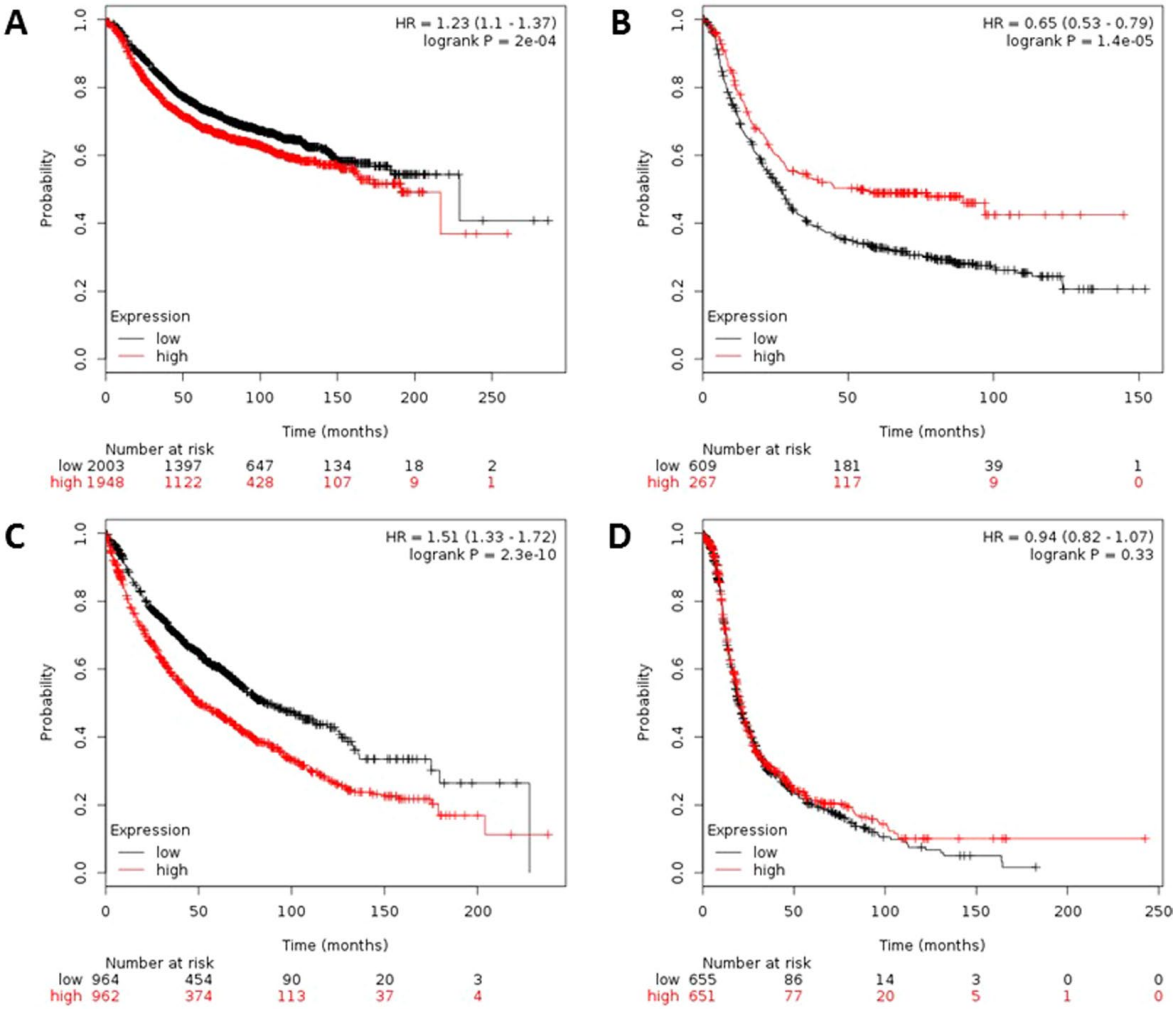
Microarray gene expression data based association of the deregulation of IL8 gene with survival of patients with different cancer types.

In a separate analysis (step 3) in our work, we attempt to associate the deregulation in IL-8 with clinical databases so as to infer possible functional consequences of IL-8 deregulation in Cancer patients. For this, we performed the Kaplan-Meier Plot analysis based on the Affymetrix microarray gene expression data from Cancer Genome Atlas (TCGA) and Gene Expression Omnibus (GEO). In the analysis, we estimated the overall survival rate of the cancer patients (breast, gastric, lungs and ovarian cancer) with IL-8 deregulation. It is widely known that the polymorphisms in deregulation of *IL8* gene render humans susceptible to inflammatory diseases (rheumatoid arthritis, inflammatory bowel diseases), cancer and visceral leishmaniasis^11–15^; however, in current study we, for the first time, associated the deregulation of the *IL8* gene with the survival of the patients with gastric and lung cancer on the basis of the results from Kaplan-Meier Plot analysis. In current study, we for the first time showed a strong correlation between IL-8 deregulation and the survival rate in cancer patients. The analysis showed that IL-8 deregulation has different implications in different cancer types, for instance, in case of gastric cancer patients the high expression of *IL8* gene is found to be associated with less number of patients at risk (more survival rate); whereas, in case of lung cancer patients the high expression of *IL8* gene is found to be associated with more patients at risk (less survival rate) (Fig. 8). Through this study, the IL-8 deregulation has also appeared to be an important prognostic marker for the detection of patients with gastric and lung cancer but not for breast and ovarian cancer. This also implicate that IL-8 deregulation does not impact the patient’s survival rate in case of sex or gender-specific cancer such as breast and ovarian cancer that are common among females. Since, SNPs are also known to deregulate the encoded protein, we believe that the 8 nsSNPs identified in this study are expected to have functional consequences similar to as in IL-8 deregulation.

## Discussion

There has been an explosion in the number of single nucleotide polymorphisms (SNPs) within public databases^52^. Single-nucleotide polymorphisms (SNPs) are considered to be the most common genetic changes that result from alterations in a single nucleotide. Among SNPs, non-synonymous SNPs (nsSNP) are associated with single amino acid substitution in the coding regions of a gene that may have the drastic effect on the structural and functional properties of the corresponding protein. These non-synonymous single nucleotide polymorphisms (nsSNPs) have been the subject of many recent studies and a large amount of data now exists in public repositories such as dbSNP^53^, HGVBase^54^ and HGMD^55^. The Swiss-Prot variant page and the ModSNP database provide a large resource for sequence and structure information on human protein variants^56^. Identification of single nucleotide polymorphisms in the coding region of a gene that have implications in inherited human diseases is the fundamental objective of research in medical genetics.

Several studies have attempted to predict the functional consequences of a nsSNP, namely whether it is disease related or neutral, based on attributes of the polymorphism. Some attributes depend only on the sequence information, for example the types of residue found at the SNP location. Single base changes in protein coding regions of DNA which lead to changes in amino acids have the potential to effect protein structure and function. In this study we focused on non-synonymous protein coding single nucleotide polymorphisms (nsSNPs), some associated with disease and others which are thought to be neutral. In our analysis, some nsSNPs are related to a disease condition but others are not associated with any change in phenotype and are regarded as neutral. A number of studies showed that the damaging/deleterious predictions scored for a particular SNP by the SIFT and PolyPhen-2 algorithms significantly correlate with the known phenotypes for that SNP based on experimental/laboratory data coming from site-directed mutagenesis studies and clinical association studies^37, 40, 57^. As found by other research groups, we also found that the combination of sequence homology based (step 1a) and structural homology based (step 1b) tools such as SIFT and PolyPhen-2 predict 70% of predicted damaging/deleterious nsSNPs correctly as damaging/deleterious^43, 57^. In current study, we have predicted the conserved amino acid residues in the IL-8 protein based on the sequence and position-specific evolutionary information using Clustal Omega and ConSurf. Structural phylogenetic analysis using ConSurf revealed that the functional residues are highly conserved in human IL-8; and most of the disease associated nsSNPs are within these conserved residues. We applied the general rule that mutations of conserved amino acid residues in a protein are deleterious while mutations of non-conserved amino acid residues are neutral. We identified the conserved amino acid residues in IL-8 protein sequence using multiple sequence alignment of IL-8 protein sequence with other phylogenetically close IL-8 sequence from other vertebrates (Fig. 3). Theoretical 3D structural models of human IL-8 were constructed using I-Tasser server and further refined and energy minimized using ModRefiner (Table 7). The structural consequences of nsSNPs can be easily ascertained by mapping nsSNPs onto the corresponding 3D structures of the wild type protein or onto the 3D structures of other proteins with high sequence similarity. If the modeled mutant proteins are different the wild type IL-8 there comes two possibilities. First is that that these mutations have destabilized the IL-8 protein or second that mutation has changed the conformation of the IL-8 protein that result in defective binding of IL-8 with its receptor CXCR1. Besides this, using stability analysis using ERIS server and docking of IL-8 wild type and mutant proteins we demonstrated that how 8 high-confidence nsSNPs affect mutant protein stability and its interaction with its receptor CXCR1. Finally, using a separate analysis (Kaplan-Meier plots) based on Affymetrix microarray gene expression data from Cancer Genome Atlas (TCGA) and Gene Expression Omnibus (GEO), we confirmed that the deregulation of *IL8* gene may results drastic affect the survival rate of gastric and lung cancer patients. The results obtained from the analysis revealed that possible alteration in structure–function relationship of IL-8 protein. The mutations caused by these 8 SNPs may have similar functional consequences as seen in case of IL-8 deregulation that suggests that these 8 SNPs could possibly lead to prioritization of diseases, such as cancer.

**Table 7:** Statistical outputs of the modeled 3D structure of different mutant IL-8 protein using I-Tasser (left side). I-TASSER predicted 3D models of mutant IL-8 proteins after structural refinement and energy minimization with ModRefiner (right side).

	Structural modelling			Refined & Energy minimized models	
Models	C-score	RSMD	TM-score	RSMD	TM-score
IL8_WT	0.57	0.79 ± 0.09	2.2 ± 1.7	0.205	0.9951
IL8_E31A	0.59	0.79 ± 0.09	2.2 ± 1.7	0.186	0.996
IL8_E31K	0.67	0.80 ± 0.09	2.0 ± 1.6	0.191	0.9958
IL8_S41Y	0.66	0.80 ± 0.09	2.1 ± 1.6	0.178	0.9963
IL8_I55N	0.61	0.80 ± 0.09	2.1 ± 1.7	0.19	0.9958
IL8_P59L	0.59	0.79 ± 0.09	2.2 ± 1.7	0.147	0.9975
IL8_P59S	0.68	0.81 ± 0.09	2.0 ± 1.6	0.211	0.9948
IL8_L70P	1.17	0.87 ± 0.07	1.2 ± 1.2	0.19	0.9958
IL8_V88D	0.61	0.80 ± 0.09	2.1 ± 1.7	0.273	0.9917

In current study, we were able to predict a high confidence data regarding the impact of amino acid substitutions on IL-8 structure and function using solely bioinformatics and computational tools. In this study, nsSNPs with associated rsIDs such as rs138567132 (E31A), rs188378669 (E31K), rs749738011 (S41Y), rs202114642 (I55N), rs765951700 (P59L), rs373821605 (P59S), rs762899923 (L70P), and rs779068762 (V88D) were found to be high-confidence mutations. We have summarized possible structural and functional consequences of nsSNPs in IL-8 protein in Table 8. We believe that further *in vitro* functional studies are required to directly determine the effect of these mutant IL-8 proteins on IL-8 binding to its receptor (CXCR1 & 2) on surface of immune cells such as neutrophils. The crystal three-dimensional structures of the mutant proteins are necessary for further validation of structural changes that have occurred as a consequence of these amino acid substitutions. Besides this, *in vitro* functional studies are also required to directly measure the binding of mutant IL-8 protein to its receptor on immune cells such as neutrophils. Whether these amino acid modifications entail any differential change in IL-8 binding to its cognate receptors, CXCR1 and 2, needs to be studied out. Additional questions related to differences in chemotactic and exocytosis activity of IL-8 mutant proteins and wild type is also need to be addressed.

**Table 8:** The summary of possible structural and functional consequences in IL-8 protein as a result of nsSNPs in *IL8* gene.

Models	Structural effect		Functional effect	
	Effect	Analysis	Effect	Analysis
IL8_E31A	No change in conformation	I-Tasser & St. superimposition	Loss of conserved functional residue	Clustalɷ & ConSurf
	Change in RSMD		Increase in protein stability	ERIS
			Increase in IL-8 binding to CXCR1	ClusPro
IL8_E31K	No change in conformation Change in RSMD	I-Tasser & St. superimposition	Loss of conserved functional residue	Clustalɷ & ConSurf
			Decrease in protein stability	ERIS
			Decrease in IL-8 binding to CXCR1	ClusPro
IL8_S41Y	No change in conformation	I-Tasser & St. superimposition	Loss of conserved functional residue	Clustalɷ & ConSurf
	Change in RSMD		Increase in protein stability	ERIS
IL8_I55N	Change in conformation Change in RSMD	I-Tasser & St. superimposition	Loss of conserved functional residue	Clustalɷ & ConSurf
			Decrease in protein stability	ERIS
IL8_P59L	Change in conformation	I-Tasser & St. superimposition	Loss of conserved functional residue	Clustal ɷ & ConSurf
	Change in RSMD		Increase in protein stability	ERIS
IL8_P59S	Change in conformation	I-Tasser &	Loss of conserved functional residue	Clustalɷ & ConSurf
	Change in RSMD	St. superimposition	Increase in protein stability	ERIS
IL8_L70P	Change in conformation Change in RSMD	St. superimposition	Loss of conserved functional residue	Clustalɷ & ConSurf
	Loss of conserved structural residue	I-Tasser	Decrease in protein stability	ERIS and MutPred
		Clustalɷ & ConSurf	Loss of catalytic residue at L70	MutPred
			Association with diseased phenotype	nsSNPAnalyzer
IL8_V88D	Change in conformation	I-Tasser & St. superimposition Clustalɷ & ConSurf	Decrease in protein stability	ERIS
	Change in RSMD		Gain of disorder	MutPred
	Loss of conserved structural residue		Loss of MoRF binding	
			Gain of ubiquitination at K91	

## Materials and Methods

### Sequence retrieval

The nucleotide sequence data on human *IL8* gene was retrieved from Entrez Gene on National Centre for Biotechnology Information (NCBI) website. The amino acid sequence of human IL8 of was collected in FASTA format from UniProt knowledgebase (UniProt ID: P10145) (http://www.uniprot.org/).

### Data mining and mapping of SNPs

All the reported SNPs of *IL8* gene were retrieved from NCBI dbSNP (http://www.ncbi.nlm.nih.gov/snp). A total of 734 SNPs were mapped in human *IL8* gene sequence. The numbers of SNPs in different functional class are reported in Fig. 1. In NCBI dbSNP database, 41 SNPs rsIDs were mapped in coding region, 100 occurred in the 3′ UTR, 21 occurred in 5′ UTR region, 137 occurred in intronic regions and the rest 435 SNPs were other human active SNPs.

### Sequence homology-based prediction of damaging coding nsSNPs using SIFT

There were 50 SNPs found associated with the 41 rsIDs mapped from dbSNP database. Prediction of deleterious effect of 50 SNPs (synonymous, non-synonymous missense and non-synonymous nonsense) in genetic variants of human *IL8* gene present on chromosome 4 was done using SIFT (http://sift.jcvi.org/). SIFT is an algorithm that predicts tolerable and intolerable (damaging) substitutions based on a sequence homology and physical properties of amino acids. The chromosome coordinates for each SNP were retrieved from dbSNP for SIFT analysis. The variants coordinates were first converted to *Homo sapiens* GRCh37 (also known as hg19) Ensembl 63 assembly/annotation version. The input format was comma separated residue based coordinate system (chromosome number, coordinate of the SNPs, orientation (1/-1), nucleotide substitution). Substitutions at a position with normalized probabilities of ≤0.05 in a tolerance index are predicted to be damaging; whereas those with normalized probabilities ≥0.05 are predicted to be tolerated^41, 58^.

### Sequence homology-based prediction of damaging coding nsSNPs using PROVEAN

PROVEAN (**Pr**otein **V**ariation **E**ffect **An**alyzer) (http://provean.jcvi.org) is a sequence based prediction tool that estimates the effect of protein sequence variation on protein function^42^. The chromosome coordinates for each SNP were retrieved from dbSNP for PROVEAN analysis. The variants coordinates were first converted to *Homo sapiens* GRCh37 (also known as hg19) Ensembl 66 assembly/annotation version. The input format was comma separated residue based coordinate system (chromosome number, coordinate of the SNPs, nucleotide substitution). A nsSNP present in the coding region of a gene is predicted to be “deleterious” if the prediction score is below threshold value (cutoff is -2.5), and “neutral” if the predicted score is above the cutoff value.

### Structural homology-based prediction of damaging coding missense nsSNPs using PolyPhen-2

PolyPhen-2 (**Poly**morphism **Phen**otyping-2) (http://genetics.bwh.harvard.edu/pph2/) is a web-based tool for annotation of coding non-synonymous SNPs. The tool employs a specific empirical rule, which comprises both physical and comparative considerations to predict the possible functional consequences of an amino acid substitution on the structure and function of a human protein. PolyPhen-2 uses query protein sequence in FASTA format as input and estimates the influence of a particular SNP or amino acid variant at a given position in query sequence^59^. The tool calculates the position-specific independent count (PSIC) score for every variant and calculates the score difference between variants.

### Identification of conserved residues and sequence motifs

The UniProt protein sequence of human IL-8 protein was BLASTed against the UniprotKB/Swiss-Prot database in NCBI (http://blast.ncbi.nlm.nih.gov/Blast.cgi) and upto maximum 100 sequences displaying significant alignment were exported as Hit Table (CSV) files. Sequences showing more than 50% identity and E-value below 1.00E-20 were chosen for further computational analysis of conserved sequences and motifs using multiple sequence alignment with Clustal Omega^60^. The amino acid identities were colored according the Clustal color scheme, and the conservation index at each alignment position were provided by Jalview^61^.

### Evolutionary phylogenetic analysis

Evolutionary conservation of amino acid in IL-8 protein was predicted by ConSurf web server^62^ by using a Bayesian algorithm (conservation scores: 1–4 variable, 5–6 intermediate, and 7–9 conserved)^63, 64^. The Clustal Omega MSA file was submitted and the conserved regions were predicted by means of coloring scheme and conservation score. Exposed and buried residues with high conservation levels were respectively scored as functional and structural residues in the human IL-8 protein sequence.

### Analysis of effect of amino acid substitution on protein stability using ERIS

ERIS server (http://troll.med.unc.edu/eris/login.php) is an *in-silico* tool for calculating mutational effect of non-synonymous amino acids substitutions on protein stability. The PDB file of the human IL-8 protein serves as an input. ERIS provides the scores for free energy alterations ΔΔG, which is equal to difference between Gibb’s free energy of mutant IL-8 protein and wild type protein (ΔG_F_ MT − ΔG_F_ WT) in Kcal/mol, where ΔΔG < 0 is indicative of decrease in stability and *vice-versa* ^65, 66^.

### Prediction of disease related amino acid substitution and phenotypes by MutPred and nsSNPAnalyzer

MutPred (http://mutpred.mutdb.org/) is an online server for prediction of molecular basis of the disease related amino acid substitution in a mutant protein^44^. It utilizes several attributes related to protein structure, function, and evolution. It uses three servers, SIFT^58^, PSI-BLAST^67^, and Pfam profiles^68, 69^, along with some structural disorder prediction algorithms, including TMHMM^70^, MARCOIL^71^, and DisProt^72^. Thus by combining the scores of all three servers, the accuracy of prediction rises to a greater extent.

The nsSNP Analyzer (http://snpanalyzer.uthsc.edu) is a web based tool to predict the phenotypic effect (disease-associated vs. neutral) of a nsSNP by using a machine learning method called RandomForest^73^. The nsSNPAnalyzer also extracts additional structural information from the SNP to facilitate the interpretation of results, for instance, structural environment, area buried, fraction polar, and secondary structure. The analysis using nsSNPAnalyzer requires information contained in the multiple sequence alignment and information contained in the input PDB 3D protein structure to make predictions.

### Structural modeling and superimposition of wild type and mutant proteins

The three-dimensional structure models of the mutant proteins were constructed using I-Tasser (http://zhanglab.ccmb.med.umich.edu/I-TASSER/), which employs an integrated combinatorial approach comprising all three standard conventional methods for structure modeling that includes comparative sequence alignment, threading, and *ab initio* modeling^74^, and predicts protein 3D structure with almost no manual intervention. Finally, energy minimization of modeled mutant proteins was carried out using ModRefiner^75, 76^. This force field permits to evaluate the energy of the modeled structure as well as overhaul distorted geometries through energy minimization. The energy minimized 3D structures of mutant IL-8 were visualized in and generated by Chimera 1.10.1. The mutant proteins were superimposed onto IL-8 wild type protein and the corresponding RSMD values were generated using generated using SuperPose ver 1.0 (wishart.biology.ualberta.ca/Superpose/)^77^.

### Molecular docking

The modeled IL-8 mutant proteins were docked onto IL-8 receptor protein, CXCR1 (PDB id: 2LNL) using ClusPro webserver (https://cluspro.bu.edu/login.php)^78^ using custom settings by entering residues in IL-8 and the receptor CXCR1 that we believe to participate in interaction. In order to specify attraction, we entered the residues corresponding to ELR^31–33^ and SGP^57–59^ for the ligand in the entry field provided in the ClusPro. The entries of the residues were in whitespace separated “chain-residue” format. For each docking experiment, the ClusPro generated 10 most-probable docking poses. The representative poses of each docking is represented in Fig. 7.

### Kaplan-Meier plotter analysis

This tool offers an excellent meta-analysis based biomarker assessment for cancer patients (http://kmplot.com/analysis)^79^. This tool is capable of examining the potential effect of 54,675 genes on survival using 10,293 cancer patient’s (comprising 5,143 breast, 1,065 gastric, 2,437 lung and 1,648 ovarian cancer patients) microarray gene expression data. Kaplan-Meier Plotter uses three versions of the Affymetrix HG-U133 datasets (with 22,277 probe sets in common), and clinical data from Gene Expression Omnibus (GEO) and The Cancer Genome Atlas (TCGA) datasets. The probe used for the *IL8* gene was ‘211506_s_at’. The overall survival analyses was run on 3951, 876, 1926, and 1306 breast, gastric, lungs, and ovarian cancer patients, respectively. Patient samples were split into two groups (high and low expression levels) based on the median value. These two groups of patients (high and low expression levels) for each breast, gastric, lung, and ovarian cancer were compared and the survival was assessed. For quality control, the biased arrays were excluded. The *p-values* below 0.05 were considered significant (Fig. 8).

## Electronic supplementary material

Supplemental Figure 1
