# Supplementary material for: Predicting the functional consequences of non-synonymous single nucleotide polymorphisms in IL8 gene
Source: Sci Rep. 2017 Jul 26;7:6525. doi: 10.1038/s41598-017-06575-4 (PMC5529537; doi:10.1038/s41598-017-06575-4)
Supplement: Supplementary file 1 — Supplemental Figure 1 [file 41598_2017_6575_MOESM1_ESM.pdf]

Predicting the functional consequences of non-synonymous single nucleotide polymorphisms in *IL8* gene

Tikam Chand Dakal[1#], Deepak Kala[2], Gourav Dhiman[2], Vinod Yadav[3],

Andrey Krokhotin[4], Nikolay V. Dokholyan[4]

[1]Department of Biosciences, Manipal University Jaipur, Dehmi Kalan, Off Jaipur-Ajmer Expressway, Jaipur-303007, Rajasthan (India)

[2]University Institute of Biopharma Sciences, Chandigarh University, Mohali-140413, Punjab (India)

[3]Department of Microbiology, Central University of Haryana, Mahendergarh-123029, Haryana (India)

[4] Department of Biochemistry and Biophysics, University of North Carolina, Chapel Hill, NC 27599, USA

Legends for Supplemental Table

Table 1S. Sequence homology-based prediction of both damaging and tolerated coding nsSNPs in *IL8* gene using SIFT and PROVEAN.

Supplemental Table S1

**SNP rsID**	**Codons**	**Substitution**	**SNP Type**	**SIFT prediction**		**PROVEAN prediction**	
				Prediction	Score	Prediction	Score
**rs572157399**	ATG-gTG	*1V	Nonsynonymous	Not scored	N/A	N/A	N/A
**rs202071309**	AAG-AcG	K4T	Nonsynonymous	Damaging	0	Neutral	-2.4
**rs563959935**	CTG-CaG	L5Q	Nonsynonymous	Damaging	0.01	Deleterious	-3.14
**rs200602609**	GCC-tCC	A6S	Nonsynonymous	Tolerated	0.42	Neutral	-0.8
**rs202202182**	GCT-GaT	A8D	Nonsynonymous	Damaging	0	Deleterious	-3.66
**rs564043731**	CTC-tTC	L9F	Nonsynonymous	Tolerated	0.39	Neutral	-2.35
**rs200254616**	CTG-CaG	L14Q	Nonsynonymous	Damaging	0	Deleterious	-4.63
**rs763622469**	GAA-GAc	E21D	Nonsynonymous	Damaging	0	Neutral	-1.47
**rs751273843**	GGT-aGT	G22S	Nonsynonymous	Tolerated	0.1	Neutral	-1.54
**rs767339386**	CCA-aCA	P26T	Nonsynonymous	Tolerated	1	Neutral	-0.25
**rs199855020**	AGT-cGT	S28R	Nonsynonymous	Damaging	0.04	Neutral	-1.13
**rs755727808**	AAA-tAA	K30*	Nonsynonymous	N/A	N/A	N/A	N/A
**rs138567132**	GAA-GcA	E31A	Nonsynonymous	Tolerated	0.07	Deleterious	-4.96
**rs188378669**	GAA-tAA	E31*	Nonsynonymous	N/A	N/A	N/A	N/A

**rs188378669**	GAA-aAA	E31K	Nonsynonymous	Damaging	0.01	Deleterious	-3.52
**rs149273289**	CAG-aAG	Q35K	Nonsynonymous	Tolerated	0.15	Neutral	-2.28
**rs149273289**	CAG-gAG	Q35E	Nonsynonymous	Damaging	0.04	Neutral	-2.17
**rs745916337**	TAC-cAC	Y40H	Nonsynonymous	Tolerated	1	Neutral	1.17
**rs144469788**	TCC-aCC	S41T	Nonsynonymous	Tolerated	0.06	Neutral	-1.37
**rs749738011**	TCC-TaC	S41Y	Nonsynonymous	Damaging	0	Deleterious	-4.27
**rs200107073**	CCT-CaT	P43H	Nonsynonymous	Tolerated	0.2	Neutral	-2.41
**rs139503118**	CAC-CgC	H45R	Nonsynonymous	Tolerated	0.12	Deleterious	-5.83
**rs774766411**	AAA-gAA	K50E	Nonsynonymous	Tolerated	0.38	Neutral	-1.02
**rs202114642**	ATT-AaT	I55N	Nonsynonymous	Damaging	0.02	Deleterious	-4.15
**rs202114642**	ATT-AcT	I55T	Nonsynonymous	Tolerated	0.39	Neutral	-1.49
**rs765951700**	CCA-CtA	P59L	Nonsynonymous	Damaging	0	Deleterious	-9.35
**rs373821605**	CCA-tCA	P59S	Nonsynonymous	Tolerated	0.3	Deleterious	-7.21
**rs147544998**	TGC-TGa	C61*	Nonsynonymous	N/A	N/A	N/A	N/A
**rs147544998**	TGC-TGt	C61C	Synonymous	Tolerated	1	Neutral	0
**rs140214046**	GCC-aCC	A62T	Nonsynonymous	Tolerated	0.39	Neutral	-0.36
**rs140214046**	GCC-tCC	A62S	Nonsynonymous	Tolerated	0.42	Neutral	0.48
**rs758228010**	ACA-cCA	T64P	Nonsynonymous	Damaging	0.01	Deleterious	-3.28
**rs142957504**	ACA-AgA	T64R	Nonsynonymous	Damaging	0.01	Deleterious	-3.53
**rs759032011**	AAG-AAa	K69K	Synonymous	Tolerated	0.38	Neutral	0
**rs759032011**	AAG-AAc	K69N	Nonsynonymous	Damaging	0.01	Neutral	0.1
**rs759032011**	AAG-AAt	K69N	Nonsynonymous	Damaging	0.01	Neutral	0.1
**rs762899923**	CTT-CcT	L70P	Nonsynonymous	Damaging	0	Deleterious	-6.4
**rs751369405**	GAG-GAc	E75D	Nonsynonymous	Damaging	0.05	Neutral	-2.02
**rs536774132**	TGT-TGa	C77*	Nonsynonymous	N/A	N/A	N/A	N/A
**rs373408845**	AAC-AAa	N83K	Nonsynonymous	Tolerated	0.31	Neutral	1.01
**rs780209935**	TGG-TGa	W84*	Nonsynonymous	N/A	N/A	N/A	N/A
**rs753921688**	AGG-AaG	R87K	Nonsynonymous	Tolerated	1	Neutral	0.63
**rs756294837**	AGG-AGa	R87R	Synonymous	Tolerated	0.67	Neutral	0

**rs756294837**	AGG-AGc	R87S	Nonsynonymous	Damaging	0.02	Neutral	-2.21
**rs779068762**	GTT-GcT	V88A	Nonsynonymous	Damaging	0	Deleterious	-3.4
**rs779068762**	GTT-GaT	V88D	Nonsynonymous	Damaging	0	Deleterious	-5.6
**rs200662278**	TTG-TaG	L93*	Nonsynonymous	N/A	N/A	N/A	N/A
**rs185040023**	AAG-AgG	K94R	Nonsynonymous	Tolerated	0.19	Neutral	-1.48
**rs200005090**	GCT-GtT	A96V	Nonsynonymous	Tolerated	0.11	Neutral	-1.3
**rs201643630**	TAA-TtA	*100L	Nonsynonymous	Not scored	N/A	N/A	N/A
